# HIV-1 active and latent infections induce disparate chromatin reorganization and transcriptional regulation of mRNAs and lncRNAs in SupT1 cells

**DOI:** 10.1128/mbio.02619-23

**Published:** 2023-12-01

**Authors:** Gabrielle Lê-Bury, Yao Chen, Jordan M. Rhen, Jennifer K. Grenier, Amit Singhal, David G. Russell, Saikat Boliar

**Affiliations:** 1 Department of Microbiology and Immunology, College of Veterinary Medicine, Cornell University, Ithaca, New York, USA; 2 A*STAR Infectious Diseases Laboratories, Agency for Science, Technology and Research, Singapore, Singapore; 3 Transcription Regulation and Expression Facility, Cornell University, Ithaca, New York, USA; University of California, Davis, Davis, California, USA

**Keywords:** HIV-1, latency, epigenome, transcriptome, lncRNA

## Abstract

HIV-1 latency is a multi-dimensional, dynamic process, and many aspects of how the viral latency is established and maintained remain incompletely characterized. We have investigated how the host cellular epigenomic and transcriptomic microenvironments compare depending on the transcriptional state of the virus. Using a dual-reporter HIV_GKO_ virus, we identified a significant divergence in chromatin reorganization and cellular gene expression patterns between active and latent HIV-1 infections of SupT1 cells. Latent HIV-1 infection induced significantly more cellular epigenomic restructuring than active infection. Furthermore, the nature of epigenomic changes also differed between the two states of HIV-1 infection. The majority of the changes in latent HIV-1 infection were repressive, whereas active viral infection led to an overall increase in chromatin accessibility. The repressed chromatin remodeling in latent infection was associated with the downregulation of genes involved in DNA replication, DNA repair, and cell cycle pathways, whereas the open chromatin structure in active infection led to increased expression of genes enriched for lipid metabolism, vesicle, and small molecule transport pathways. Similar to cellular mRNAs, our comprehensive transcriptomic analysis also identified a significant shift in long non-coding RNA (lncRNA) epigenomic and expression profiles between active and latent HIV-1 infections. Putative functional roles of novel lncRNAs associated with cellular pathways dysregulated in active and latent HIV-1 infections were identified, and the effects of viral reactivation on lncRNA expression in latently infected cells were evaluated. This study revealed that HIV-1 latent infection necessitates substantially greater alterations in the cellular epigenome and transcriptome than active HIV-1 infection.

**IMPORTANCE:**

HIV-1 infection of T-lymphocytes depends on co-opting cellular transcriptional and translational machineries for viral replication. This requires significant changes in the cellular microenvironment. We have characterized and compared the changes in cellular chromatin structures as well as gene expression landscapes in T cells that are either actively or latently infected with HIV-1. Our results reveal that chromatin accessibility and expression of both protein-coding mRNAs and non-coding lncRNAs are uniquely regulated in HIV-1-infected T cells, depending on whether the virus is actively transcribing or remains in a transcriptionally silent, latent state. HIV-1 latent infection elicits more robust changes in the cellular chromatin organization than active viral infection. Our analysis also identifies the effects of such epigenomic changes on the cellular gene expression and subsequent biological pathways. This study comprehensively characterizes the cellular epigenomic and transcriptomic states that support active and latent HIV-1 infection in an *in vitro* model of SupT1 cells.

## INTRODUCTION

One major challenge in the cure for HIV-1 infection is the persistence of latently infected cells. T lymphocytes and macrophages can both serve as viral reservoirs, but resting memory CD4^+^ T cells are considered the main cell type for HIV-1 latency (1–5). Current combination anti-retroviral therapy (cART) can effectively impede viral replication but cannot eliminate latently infected reservoir cells. Even after prolonged cART, the virus rebounds rapidly from persistently infected reservoirs upon treatment interruption. The mechanisms of HIV-1 latency are not completely understood, but it is evident that transcriptional silencing of the provirus is multifactorial and regulated at both viral and cellular levels (6, 7). For instance, epigenetic repression of the proviral promoter blocks viral transcription (8–10). The site of viral integration into the host genome is another determinant of viral gene expression (11). Insufficiency of cellular transcription factors, e.g., NF-kB, NFAT, and P-TEFb, can also result in viral latency (12, 13).

HIV-1 replication in infected cells relies on the host cellular machinery. Therefore, a comprehensive understanding of the cellular microenvironments that support the establishment and maintenance of viral replication or latency is crucial for designing effective curative interventions for HIV-1. Multiple studies have undertaken epigenomic, transcriptomic, and proteomic analyses of HIV-1-infected cells, both *in vivo* and *in vitro*, to interrogate the cellular and molecular changes during active or latent viral infections (14–17). HIV-1 can modulate host defense pathways such as innate or adaptive immune response and immune activation at both epigenomic and transcriptomic levels (18, 19). Differential usage of cell metabolic pathways is also evidenced in active or latent infection (20).

HIV-1 is a master regulator of cellular processes. Apart from protein-coding mRNAs, HIV-1 infection also modulates the expression of various non-coding RNAs such as microRNAs and long non-coding RNAs (lncRNAs) (21, 22). In recent years, numerous lncRNAs have been identified with key roles in HIV-1 replication and pathogenesis. A group of lncRNAs, including *Malat1*, *HEAL*, *NRON*, and *NEAT1*, have been proven crucial in regulating HIV-1 replication and latency (23–26). On the other hand, lncRNAs *SAF* and *lincRNA-p21* can promote viral persistence by promoting the survival of the infected cells (27, 28). Despite these multifaceted studies, a knowledge gap exists. Recent technological advancements in high-throughput analysis and its usage in unbiased comparison of active and latently infected cells can help advance our understanding of the specific molecular pathways that shape the active and latent HIV-1 infections.

The absence of definite cellular markers for latently infected cells as well as their rarity *in vivo* greatly hinders the in-depth study of viral reservoir cells obtained directly from HIV-1-infected individuals. Most of the advancements in our fundamental understanding of the mechanisms of HIV-1 latency have been achieved with *in vitro* models. Several *in vitro* models of HIV-1 latency are currently available, and their merits and limitations have been discussed extensively (29).

In this study, we used a single-round, dual-reporter HIV-1 virus, HIV_GKO_, which encompasses two distinct fluorescent readouts: one for viral infection and provirus integration (mKO2 under cellular promoter EF1α) and another to indicate active viral transcription (GFP under HIV-1 LTR promoter) (30). Infection with HIV_GKO_ provides a distinct advantage of simultaneous identification of actively infected (viral transcription-active mKO2^+^GFP^+^) and latently infected (viral transcription-inactive mKO2^+^GFP^-^) cells from within a pool of infected cells. We have utilized this *in vitro* model of HIV-1 infection in SupT1 cells and carried out an integrated, comparative analysis of the active and latently infected cells to that of virus-uninfected cells to gain insights into the epigenomic and transcriptional landscapes that distinguish the two states of HIV-1 infection.

In this study, the assay for transposase-accessible chromatin with sequencing (ATAC-seq) data revealed a drastic repression of cellular chromatin accessibility in latently infected cells, while active HIV-1 infection enhanced the overall accessibility of the chromosomes. Consequently, there was a greater degree of modulation of gene expression, as revealed by RNA-seq, in latent infection that affected biological pathways such as RNA metabolism, transcription, and cell cycle. Through guilt-by-association analysis, we also identified several novel lncRNAs with potential roles in HIV-1 replication and latency. Viral reactivation in latent cells reverted the expression of latency-associated lncRNAs toward the levels of active infection. Overall, our study revealed that latent HIV-1 infection has a greater impact than active infection on cellular chromatin organization and gene expression in T cells.

## RESULTS

### HIV-1 latent infection elicits markedly repressed reorganization of the cellular chromatin

**Fig 1:**
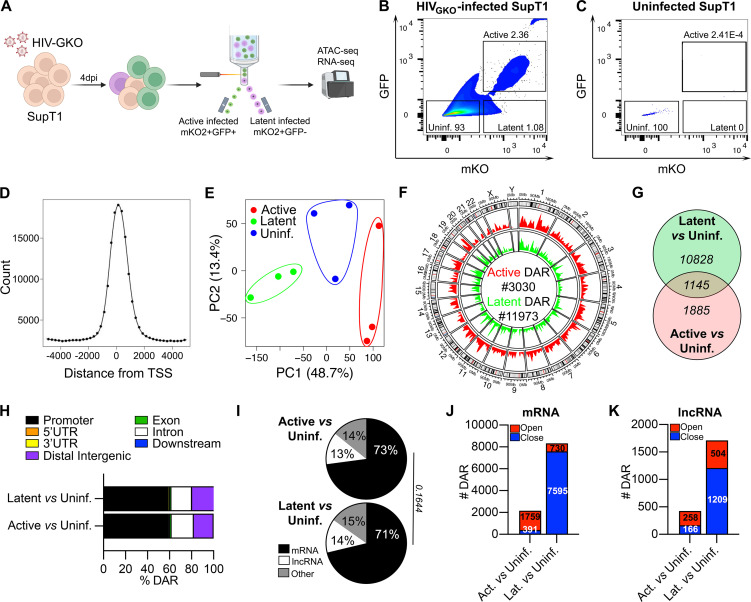
Chromatin accessibility in active and latent HIV-1 infections of SupT1 cells. (**A**) Schematic diagram of experimental design. (**B and C**) Representative flow-cytometry plots showing the gating strategy for sorting HIV-1 active and latently infected (**B**) and virus non-exposed uninfected (**C**) SupT1 cells. (**D**) TSS enrichment plot of ATAC-seq peaks for combined data sets of active, latent, and uninfected SupT1 cells. (**E**) PCA plot of ATAC-seq peaks from uninfected (blue), active-infected (red), and latent-infected (green) SupT1 cells. (**F**) Circos plot showing the distribution of differential accessible regions (DARs) (fold change > 1.5, FDR < 0.05) across the chromosomes of the human genome. Histograms represent the numbers of peaks for active-infected (red) and latent-infected (green) SupT1 cells. Number of open (red) and closed (blue) DARs (FDR < 0.05, fold change > 1.5) in active infection (left) or latent infection (right) compared to uninfected SupT1 cells. (**G**) Venn diagram of common or exclusive DARs for active (red) or latent (green) infection compared to uninfected SupT1 cells. (**H**) Stacked bar plots indicating the genomic feature distributions of DARs in active (bottom) and latent (top) infections compared to uninfected SupT1 cells. Promoter (black), 5′ UTR (orange), 3′ UTR (yellow), distal intergenic (purple), exon (green), intron (white), and downstream (blue) are color-coded. (**I**) Percentages of DARs associated with mRNAs (black), lncRNAs (white), and other transcripts (gray) in active (left) and latent (right) infections compared to uninfected SupT1 cells. Chi-square test was used to compare the distribution of frequencies. (**J and K**) Numbers of DARs (FDR < 0.05, fold change > 1.5) associated to mRNAs (**J**) and lncRNAs (**K**) that are open (red) or closed (blue) in active (left) and latent (right) infections compared to uninfected SupT1 cells.

HIV-1 latency in CD4^+^ T cells is characterized by transcriptional silencing of viral genes, which is often attributed to limited transcription factor accessibility to the proviral promoter (31). To investigate whether such epigenomic alterations also extend to the cellular genome, we compared the host chromatin architecture in active and latently HIV-1-infected SupT1 cells to that of uninfected cells. HIV_GKO_-infected SupT1 cells were flow-sorted 4 dpi into the two populations: active (mKO2^+^GFP^+^) and latent (mKO2^+^GFP^-^) HIV-1-infected cells (Fig. 1A and B). Virus non-exposed cells were flow-sorted as uninfected controls (Fig. 1C). Flow-sorted cells from three independent experiments were processed for ATAC-seq. We obtained an average of 49,000 peaks per sample and a total of 131,019 peaks across all samples. The distribution of chromatin accessibility peaks was comparable among the samples with most peaks located near transcription start sites (TSS) (Fig. 1D). Furthermore, principal component analysis (PCA) revealed separation of cells based on infection status, suggesting active and latent infection-specific chromatin organization (Fig. 1E).

Comparison of chromatin accessibility profiles of the infected cells against uninfected ones revealed that 3,030 and 11,973 annotated regions of the genome were significantly differentially accessible (DAR, differential accessible regions, fold change > 1.5, FDR < 0.05) in active- and latently infected cells, respectively (Fig. 1F). This indicates that latent HIV-1 infection leads to significantly greater (approximately fourfold) chromatin reorganization than active infection. Although the DARs were similarly distributed across different chromosomes of the human genome in both infected cell types (Fig. 1F), only 1,145 genomic regions shared altered accessibility between active and latent infections in comparison to uninfected cells (Fig. 1G). The distribution pattern of DARs across different gene regulatory regions was also similar in both infected cells. The majority of DARs were associated with promoter regions (TSS ± 3 kb) of genes, accounting for about 59% of DARs in both active and latent infections (Fig. 1H). Next, to understand if the chromatin changes differentially affected protein-coding mRNAs and non-coding lncRNAs, we classified the DARs based on their association with known annotated genes. The proportion of DARs impacting either mRNAs (73% versus 71%) or lncRNAs (13% versus 14%) remained comparable in both infected cell types (Fig. 1I). However, the directionality of epigenomic changes showed drastic opposing patterns in active and latent infections, for both mRNAs and lncRNAs. In active HIV-1 infection, the proportions of open DARs in mRNAs and lncRNAs were almost 4.5-fold and 1.6-fold higher than closed ones, respectively (Fig. 1J and K). In contrast, in latently infected cells, the number of closed regions associated with mRNAs and lncRNAs far exceeded (10.4-fold and 2.4-fold, respectively) the number of open DARs (Fig. 1J and K). Together, our data depict an overall increased chromatin accessibility in active HIV-1 infection, whereas latent infection led to a collective decrease in chromatin accessibility. This indicates that HIV-1 latent infection in SupT1 cells triggers a more extensive, largely repressive reorganization of the cellular chromatin than in actively HIV-1-infected cells.

### HIV-1 latent infection induces broader cellular transcriptomic changes than active infection

**Fig 2:**
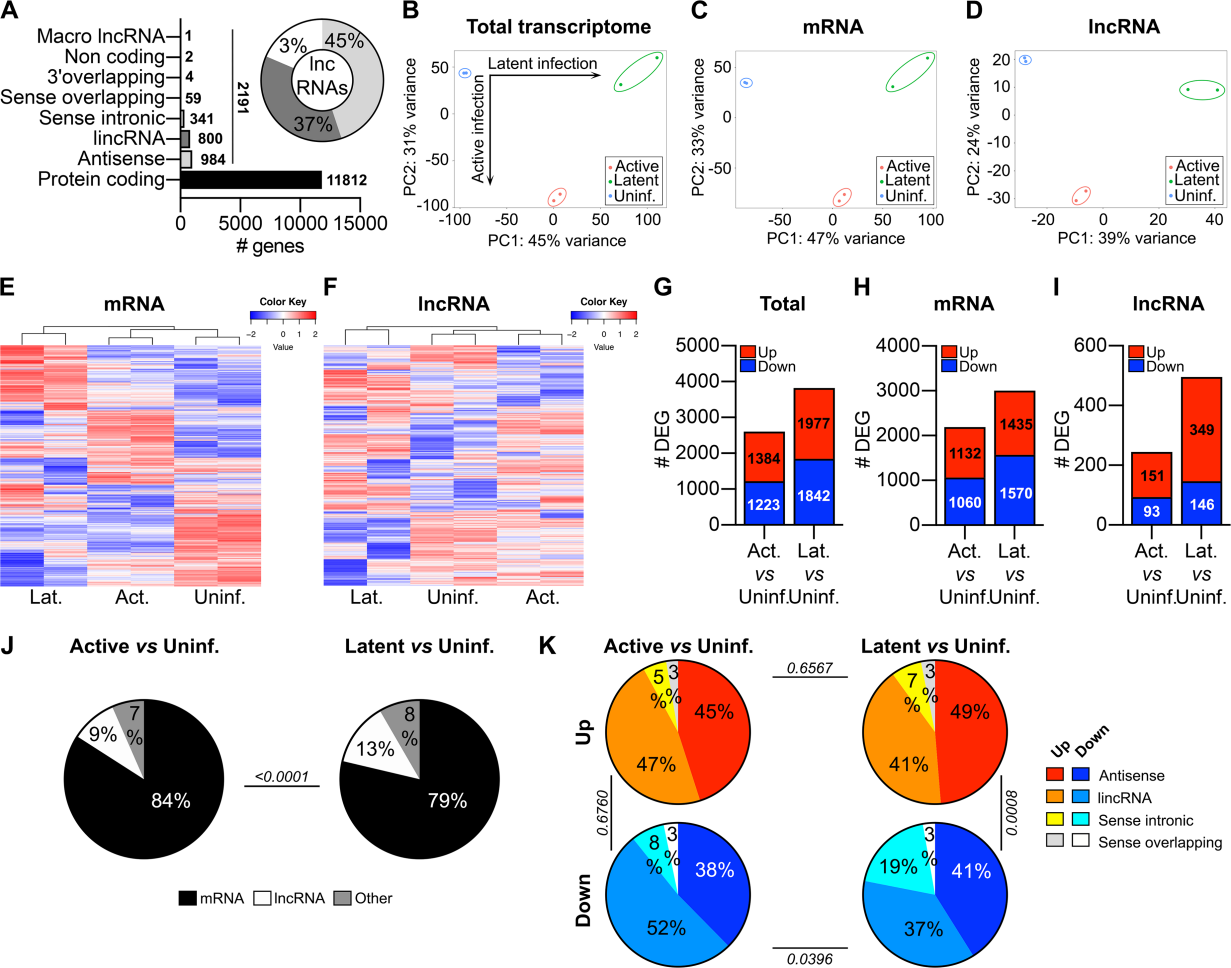
Global transcriptomic analysis of active and latent HIV-1-infected SupT1 cells. (**A**) Distribution of transcript biotypes identified (cpm > 1 in at least 50% of samples) in RNA-seq data sets. Diagram shows the proportions of lncRNA subclasses. (**B–D**) PCA plots representing the total transcriptome (**B**), mRNAs (**C**), and lncRNAs (**D**) expression profiles in uninfected (blue), active-infected (red), and latent-infected (green) SupT1 cells. (**E and F**) Heat maps of all detected mRNAs (**G**) and lncRNAs (**H**) expression. (**G–I**) Numbers of DEGs (FDR < 0.05, fold change > 2) for total transcripts (**G**), mRNAs (**H**), and lncRNAs (**I**) that are upregulated (red) or downregulated (blue) in active (left) and latent (right) infections compared to uninfected SupT1 cells. (**J**) Percentages of mRNAs (black), lncRNAs (white), and other transcripts (gray) that are differentially expressed in active (left) and latent (right) infections compared to uninfected SupT1 cells. Chi-square test was used to compare the distribution of frequencies. (**K**) Percentages of lncRNA subclasses that are upregulated (top) or downregulated (bottom) in active (left) and latent (right) infections compared to uninfected SupT1 cells. Chi-square test was used to compare the distribution of frequencies.

Since the majority of DARs were in the promoter regions of genes (Fig. 1H), we investigated how the chromatin reorganization affected the cellular transcriptome during the two states of HIV-1 infection. RNA sequencing was performed on uninfected, active- and latently infected SupT1 cells from two independent experiments. Expression of a total of 15,724 RNA transcripts was detected across the samples, of which 11,812 were mRNAs and 2,191 were lncRNAs (Fig. 2A). Among different subclasses, the majority of the expressed lncRNAs belonged to lincRNAs and antisense lncRNAs, accounting for 45% and 37%, respectively (Fig. 2A).

Next, we determined how the two states of HIV-1 infection affected the cellular transcriptomes. Unsupervised PCA analysis of the total transcriptomes showed robust segregation among the three different cell populations based on their infection states (Fig. 2B). We then separated the mRNAs and lncRNAs and analyzed their expression profiles either by PCA or unbiased hierarchical clustering to understand their relative contributions to the divergent host response in the two infected cell types (Fig. 2C and D). These analyses revealed that the distinctions between uninfected and active or latently infected cells were accurately reflected in the expression of both lncRNAs and mRNAs. Interestingly, for both mRNAs and lncRNAs, latently infected cells clustered on a distinct branch on the dendrogram, depicting a higher degree of transcriptomic reprogramming in latent than active infection (Fig. 2E and F). These expression profiles indicate that active and latent HIV-1 infections impart unique transcriptional signatures for both mRNAs and lncRNAs that effectively distinguish the state of viral infection.

To measure the magnitude of host transcriptional changes during HIV-1 infection of SupT1 cells, we identified the differentially expressed (DE, fold change > 2 and FDR < 0.05) RNA transcripts in active and latently infected cells compared to uninfected ones. A total of 2,607 genes were DE during active infection compared to 3,819 genes in latent, indicating broader host transcriptomic changes in HIV-1 latency than active infection (Fig. 2G). The pattern was similarly reflected in both DE mRNAs and lncRNAs, although the difference was greater for lncRNAs with twice as many DE lncRNAs in latent than in active infection (Fig. 2H and I). Furthermore, DE lncRNAs accounted for a larger portion (13%) of total dysregulated genes in latent compared to active (9%) infection (Fig. 2J). Comparing the directionality, the number of up- or downregulated mRNAs was proportionate within each group of virus-infected cells (Fig. 2H). However, a considerably higher number of lncRNAs were upregulated than downregulated in both states of infection (active: 1.6-fold, latent: 2.4-fold; Fig. 2I). The distributions of different classes of DE lncRNAs were mostly similar between active and latent infections, except a significantly higher proportion of sense intronic lncRNAs were downregulated in latent infection (Fig. 2K). Overall, these data show a greater dynamic shift in cellular transcriptomes during latent infection than in active HIV-1 infection.

### Dichotomous relation between chromatin reorganization and gene expression in active and latent HIV-1 infections

**Fig 3:**
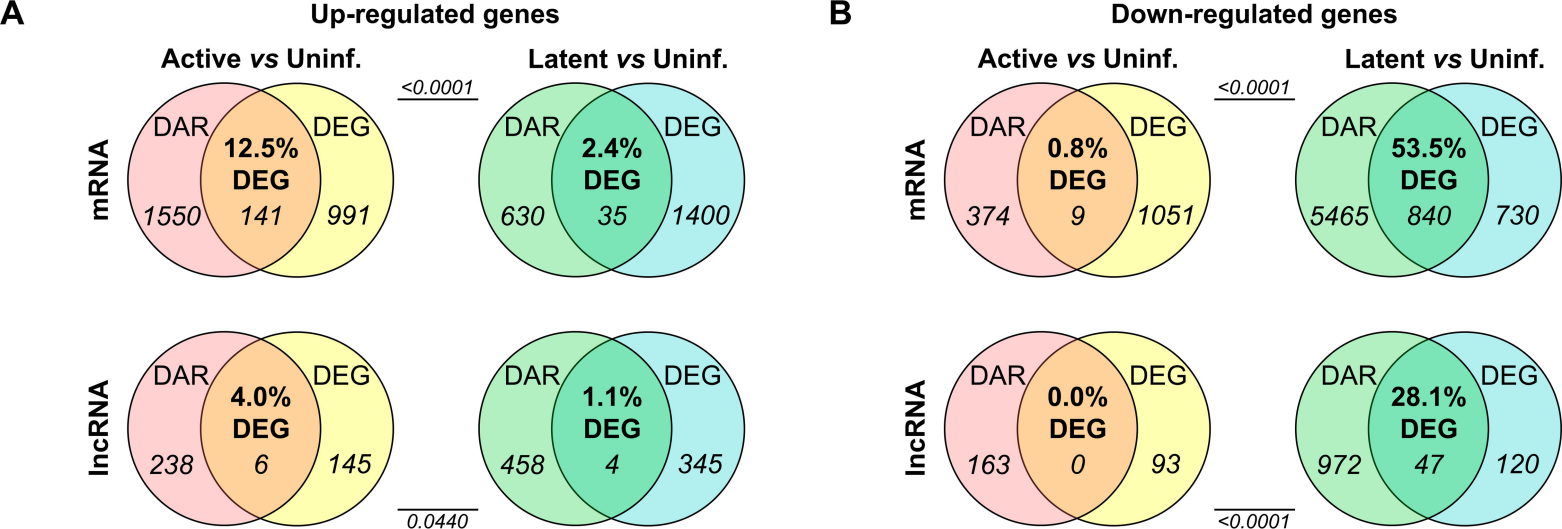
Correlation between chromatin accessibility (ATAC-seq) and expression (RNA-seq) of mRNAs and lncRNAs in HIV-1-infected SupT1 cells. (**A and B**) Venn diagrams of significantly upregulated (**A**) and downregulated (**B**) DEGs and DARs, corresponding to mRNAs (top) and lncRNAs (bottom) in active (left) and latent (right) infections compared to uninfected SupT1 cells. Chi-square test was used to compare the distribution of frequencies.

To understand the contributions of chromatin accessibility changes onto transcription of corresponding genes in HIV-1 active- and latently infected SupT1 cells, we integrated ATAC-seq and RNA-seq data sets to estimate the number of up- and downregulated genes that were mutually enriched in both DARs and DEGs. Associations between changes in gene expression and corresponding genomic region chromatin accessibility showed a disparate pattern based on the directionality of transcriptional changes and the state of HIV-1 infection. When segregated based on the directionality of changes, for upregulated genes (both mRNAs and lncRNAs), a significantly greater proportion of DEGs had a corresponding DAR with increased accessibility in actively infected than in latently infected cells (Fig. 3A and B). This indicates that gain in chromatin accessibility plays a greater role in increased gene expression in active infection. In contrast, a significantly higher fraction of downregulated DEGs (both mRNAs and lncRNAs) had a corresponding repressed DAR in latently infected cells than in active infection (Fig. 3A and B). These data reveal that the restriction of chromatin accessibility is a major contributor to the downregulation of cellular gene expression in latent HIV-1 infection. However, the overall association between epigenomic changes and cellular gene expression was significantly higher in latent than active infection, suggesting a greater role of chromatin reorganization in the regulation of gene expression in latently infected cells (Fig. 3A and B). These data imply an inherent difference in chromatin remodeling-mediated regulation of gene expression in active- and latently infected SupT1 cells, wherein a greater degree of correlation exists between chromatin remodeling and gene expression in latent HIV-1 infection.

### Discrete effects of active and latent HIV-1 infections on cellular pathways

**Fig 4:**
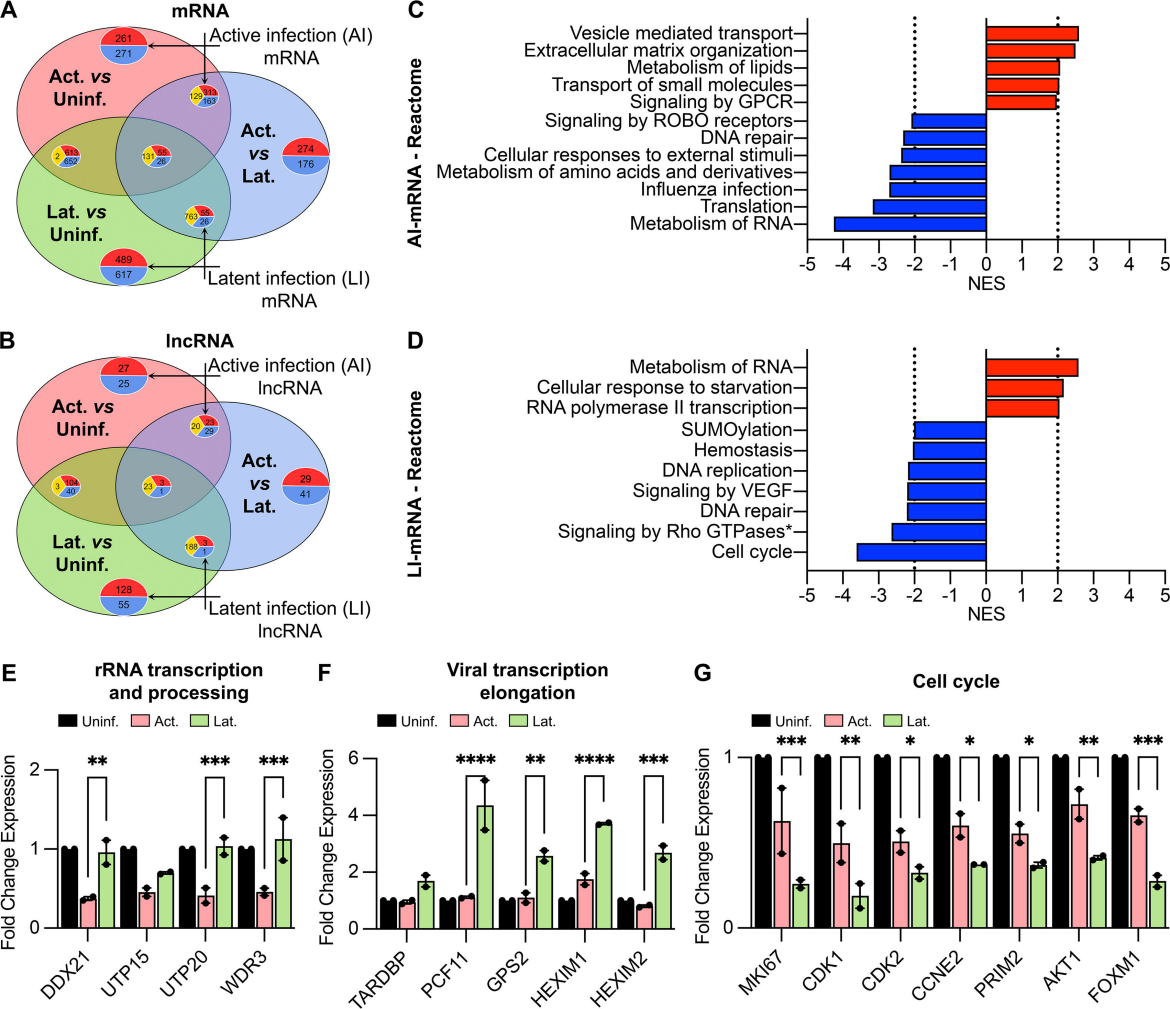
Biological pathways enriched in active and latent HIV-1 infection of SupT1 cells. (**A and B**) Venn diagrams of DEGs (FDR < 0.05, fold change > 2) among “Active versus Uninfected” (light red), “Latent versus Uninfected” (light green), and “Active versus Latent-infected” (light blue) SupT1 cells for mRNAs (**A**) and lncRNAs (**B**). Numbers of DEGs that are upregulated (red), downregulated (blue), or contraregulated (yellow) are indicated within the plots.(**C and D**) Biological pathways enriched (NES > 2) within the Reactome database for DE mRNAs (FDR < 0.05, fold change > 2) specific to AI-mRNA (**C**) and LI-mRNA (**D**) (*signaling by Rho GTPases, Miro GTPases, and RHOBTB3). (**E–G**) Relative fold change in the expression of genes involved in rRNA transcription and processing (**E**), viral transcription and elongation (**F**), and cell cycle (**G**) for active (red) and latent (green) infections compared to uninfected (black) SupT1 cells. Mean ± SEM is presented, and two-way ANOVA Fisher’s LSD test was used to calculate *P*-values (*P < 0.03; **P < 0.002; ***P < 0.0002; and ****P < 0.0001).

We next investigated the biological pathways that were specifically impacted in active- and latent-infected SupT1 cells. We first sought to identify the mRNAs and lncRNAs that showed unique expressions in either active or latent infection and excluded the ones that were mutually altered in both HIV-1 infection states. We analyzed the DE gene sets with a proportional Venn diagram to identify the unique and shared transcripts across the cell populations (Fig. 4A and B). RNA transcripts that were exclusively DE in active infection versus uninfected cells, as well as the genes that were mutually up- or downregulated in active infection compared to both uninfected and latent-infected cells, were considered active infection-specific (AI-mRNA or AI-lncRNA), as depicted in Fig. 4A and B. Similarly, DE genes that were unique to latently infected cells compared to either active or uninfected cells were designated latent infection-specific (LI-mRNA or LI-lncRNA) (Fig. 4A and B). Based on these criteria, we identified 1,008 mRNAs and 104 lncRNAs that were specific to active infection. A larger pool of 1,869 mRNAs and 371 lncRNAs were found to be altered exclusively in latent HIV-1 infection.

To understand which biological processes are impacted uniquely in active and latently HIV-1-infected SupT1 cells, we performed pre-ranked gene set enrichment analysis (GSEA) with the active and latent infection-associated mRNAs using the Reactome database of canonical pathways. Pathways with a normalized enrichment score (NES) > 2 and *P*-value < 0.01 were considered significantly altered (Fig. 4C and D). Cellular transport of small molecules and vesicular cargo was markedly increased in actively infected cells. Active viral replication also upregulated G-protein coupled receptor-mediated signal transduction (Fig. 4C). On the other hand, pathways associated with DNA repair, responses to external stimuli, and metabolism of amino acid derivatives were downregulated in actively infected cells (Fig. 4C). Interestingly, biological pathways associated with the metabolism of RNAs showed opposite regulation in active versus latent infection (Fig. 4C and D). In latently infected cells, RNA metabolic processes along with RNA polymerase II-mediated transcription were upregulated, whereas DNA damage response, DNA replication, and cell cycle pathways were significantly downregulated (Fig. 4D).

RNA metabolic processes were suppressed in active infection but upregulated in latent infection. Metabolism of RNA is a super-pathway that encompasses important biological processes including the conversion of nascent RNA transcripts into mature mRNAs (capping, splicing, and polyadenylation) as well as the editing and decaying of mRNAs. In active HIV-1 infection, rRNA processing was the predominant subnetwork that was significantly downregulated, as a majority (36/70 genes) of the RNA metabolism-enriched genes were involved in this process. Of note, HIV-1 infection of T lymphocytes impairs ribosome biogenesis by inhibiting pre-rRNA processing (15). Genes related to rRNA transcription and processing, such as *DDX21*, *UTP15*, *UTP20*, and *WDR3*, were downregulated in actively infected cells (Fig. 4E). Consistent with the inhibition of ribosomal biogenesis, active viral replication also led to the suppression of the host translational machinery (Fig. 4C). Conversely, in latently infected cells, RNA polymerase II-dependent transcription and metabolic processing of mRNAs were elevated compared to uninfected cells (Fig. 4D).

Intriguingly, it is known that one of the main contributing factors to HIV-1 latency is the suppression of viral transcription. Our data suggest that inhibition of HIV-1 viral transcription in latently infected cells is independent of overall suppression of cellular transcription. Although the relative increase in cellular transcription observed in latently infected cells could be due to a decrease in cellular transcription and translation in actively infected cells. HIV-1 replication is known to inhibit these cellular processes. However, the expression of several transcription factors that enhance cellular transcription, such as zinc finger proteins (*ZNF470* and *ZNF573*) and mediator complex subunits (*MED6, MED26*, and *MED31*), was increased in latently infected SupT1 cells (data not shown). On the other hand, suppressors of viral transcription, such as *TARDBP*, which inhibits viral transcription by binding to the HIV-1 TAR element (32), were increased (1.7-fold) in latent infection (Fig. 4F). Furthermore, repression of HIV-1 gene expression is often attributed to promoter-proximal stalling of RNA polymerase II and premature termination of viral transcription by increased recruitment of cleavage and polyadenylation factors to the viral transcription start site. The expression of several factors implicated in the inhibition of viral transcription elongation, such as *PCF11* (4.3-fold), *GPS2* (2.6-fold), *HEXIM1* (3.7-fold), and *HEXIM2* (2.7-fold), was significantly upregulated in latent infection compared to actively infected cells (Fig. 4F). Our results demonstrate that inhibition of HIV-1 transcription in latently infected SupT1 cells is virus-specific and not part of a global downregulation in cellular transcription or post-transcriptional processes.

DNA replication and cell cycle pathways were markedly downregulated in latent infection. Biological pathway analysis also showed a significant decrease in cell proliferation pathways in latently infected SupT1 cells. Some differentially enriched pathways within the cell cycle included cell cycle checkpoints, resolution of sister chromatid cohesion, and mitotic spindle checkpoints. The cell proliferation marker *MKI67* as well as genes involved in G1-S and G2-M transition, such as *CDK1*, *CDK2*, *CCNE2*, *PRIM2*, *AKT1*, and *FOXM1*, were significantly downregulated in latent infection compared to actively infected cells (Fig. 4G). HIV-1 accessory protein, Vpr, is known to impede the cell cycle (33, 34) and accordingly, we observed a trend of lower expression of the cell cycle genes in actively infected cells compared to uninfected ones (Fig. 4G). However, the reductions were significantly more in latently infected cells (Fig. 4G), indicating a possible Vpr-independent cell cycle arrest in latent HIV-1 infection.

### Identification of putative associations of lncRNAs to cellular pathways dysregulated in active and latent HIV-1 infections

**Fig 5:**
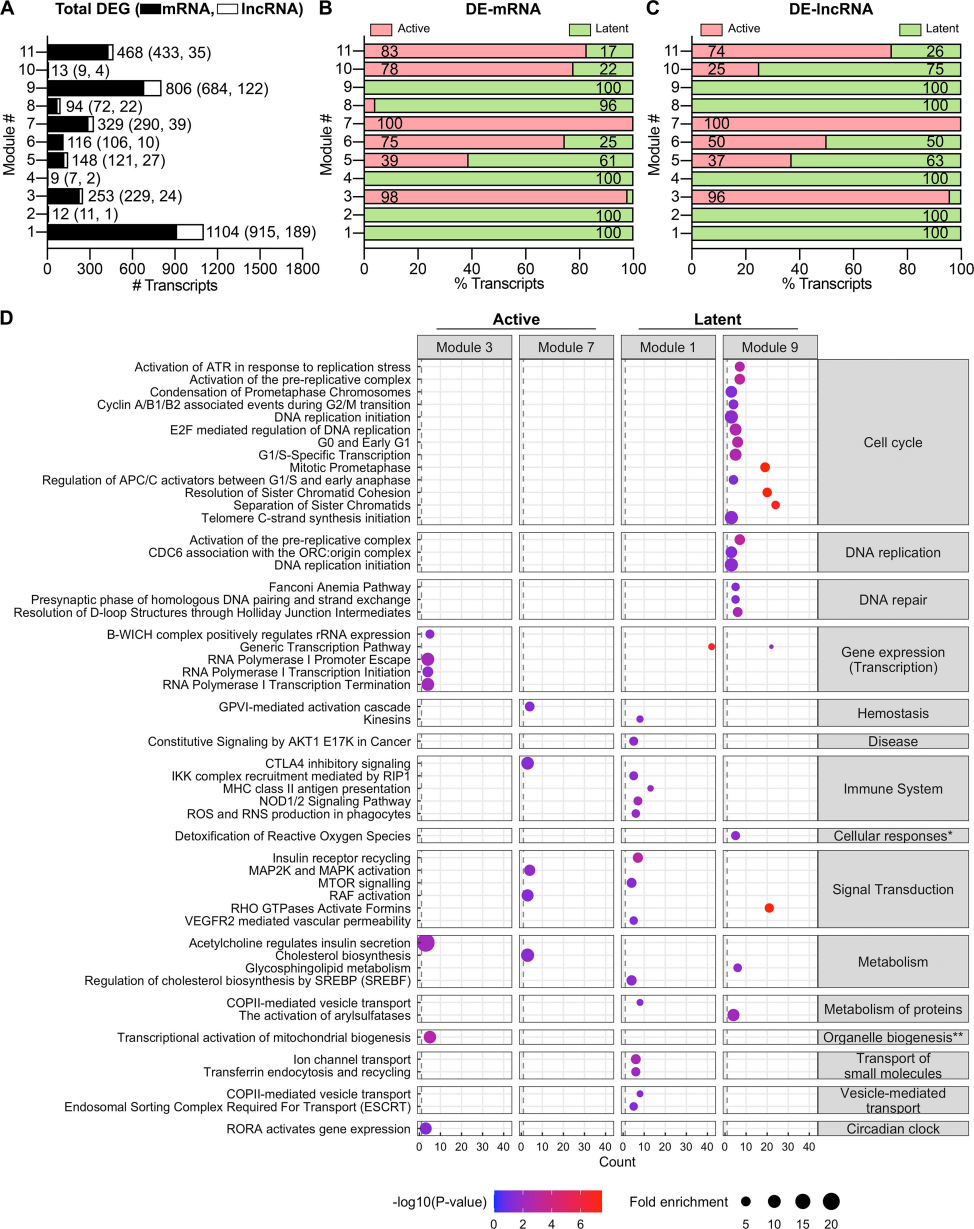
Identification of putative functions of DE-lncRNAs in active and latent HIV-1 infections of SupT1 cells. (**A**) Number of transcripts in each module identified by WGCNA for mRNAs (black) and lncRNAs (white). (**B and C**) Percentages of DE-mRNAs (**B**) and DE-lncRNAs (**C**) in active (red) and latent (green) infections within each module. (D) Significantly enriched (*P*-value < 0.05) pathways within the Reactome database for active infection-specific modules 3 and 7 and in latent infection-specific modules 1 and 9. The dot size and color represent fold enrichment of the pathways and their significance [−log(*P*-value)], respectively (*Cellular responses to external stimuli and **Organelle biogenesis and maintenance).

To gain an insight into the biological functions of the DE lncRNAs, most of which are functionally undefined, we used a guilt-by-association analysis and constructed co-expression networks of mRNAs and lncRNAs. We analyzed all detectable RNA transcripts in the data set by unsupervised weighted gene co-expression network analysis (WGCNA) for hierarchical clustering using the “unsigned” mode to account for the opposing regulations that are frequently observed between interacting lncRNAs and mRNAs. A total of 11 modules of significantly correlated transcripts were detected (Fig. 5A). Interestingly, mRNAs and lncRNAs did not cluster separately but rather remained coalesced within each module (Fig. 5A). However, active- and latent-infection-associated genes remained mostly segregated into different modules, further supporting the transcriptional divergence in these two states of HIV-1 infection (Fig. 5B and C). Modules 1, 2, 4, 8, and 9 included latency-associated genes, although the vast majority of the latency-specific mRNAs and lncRNAs were contained in modules 1 and 9 (Fig. 5A through C). In contrast, a substantial fraction of active infection-specific genes was assigned to modules 3 and 7 (Fig. 5A through C). Four modules (5, 6, 10, and 11) were populated with a mix of genes linked to both active and latent HIV-1 infections. We, therefore, focused our subsequent analysis on four major modules that encompassed the bulk of either active infection (modules 3 and 7) or latent infection (modules 1 and 9) associated genes.

A mutual expression pattern between mRNAs and lncRNAs often suggests shared biological functions and/or regulatory relations. To understand the potential functions of the lncRNAs, we analyzed the member mRNAs within the active or latent infection-associated modules for enrichment in canonical pathways based on the Reactome database (Fig. 5D). Module 3, which contained predominantly active infection-specific genes, was enriched in annotations related to gene transcription pathways such as RNA polymerase I-mediated transcription initiation and termination as well as transcription of rRNAs and mitochondrial genes (Fig. 5D). This suggests that the lncRNAs in module 3 could possibly be involved in RNA metabolism and translation, two processes that were found to be significantly downregulated in actively infected cells (Fig. 4C). The active-infection-associated module 7 showed enrichment for pathways linked to immune responses, signal transduction, and cholesterol metabolism. Metabolism of lipids was also one of the pathways that were found to be upregulated in actively infected cells (Fig. 4C). This contrasts with latent infection-associated modules where a large number of mRNAs were associated with pathways of gene transcription in module 1 and cell cycle, DNA replication, or repair in module 9 (Fig. 5D). Notably, gene transcription was upregulated and cell cycle, DNA replication, and DNA damage pathways were found downregulated in latently infected cells (Fig. 4D).

**Fig 6:**
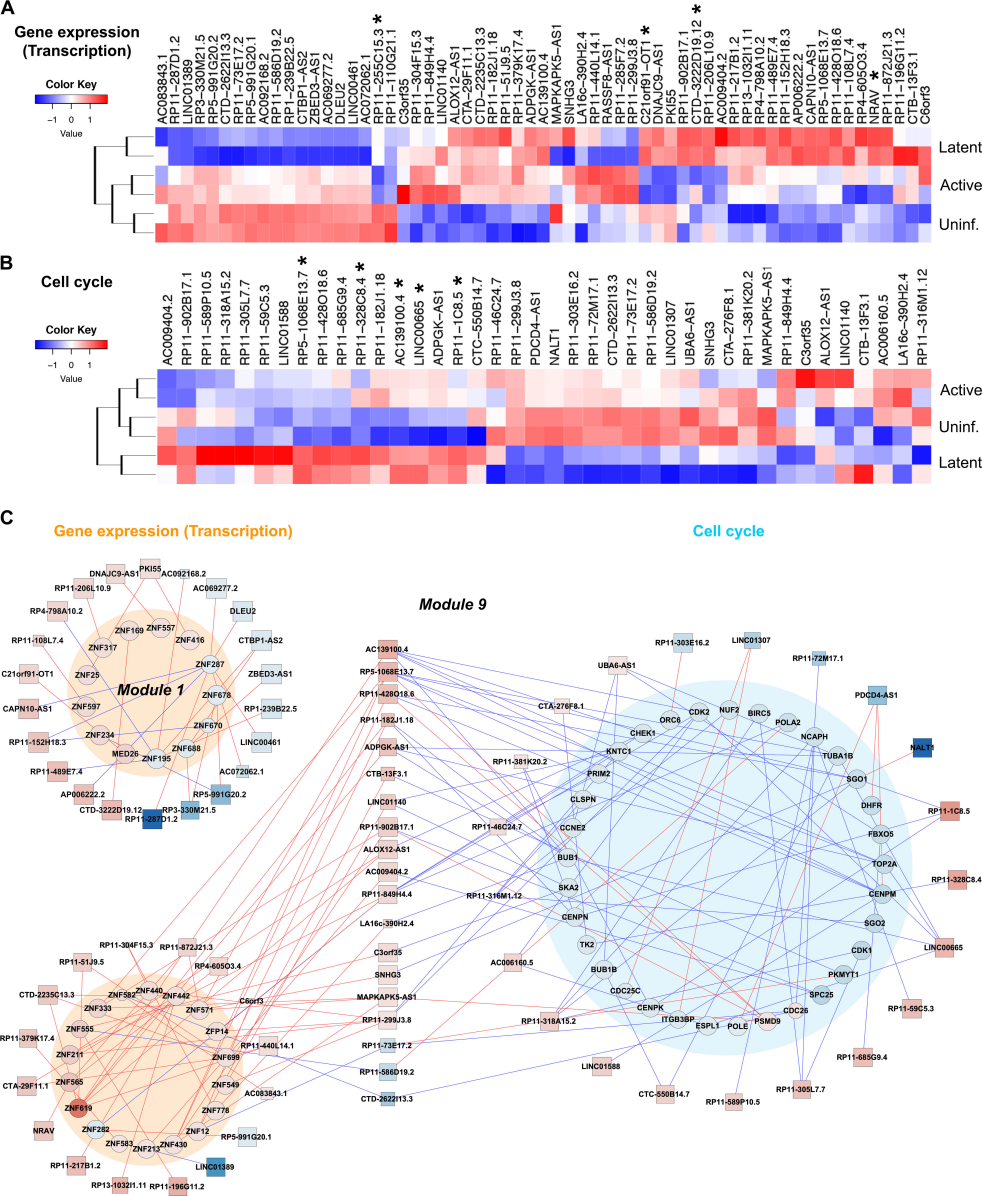
RNA transcription and cell cycle-associated lncRNAs (A and B). Heat maps of the expression of lncRNAs associated with RNA transcription (**A**) and cell cycle pathways (**B**). Asterisk represents lncRNAs selected for reactivation study (Fig. 7C and D). (**C**) Cytoscape correlation networks within RNA transcription (orange circles) and cell cycle pathways (blue circle) of upregulated (red) and downregulated (blue) mRNAs (circle) and lncRNAs (square) with Pearson correlation coefficient > 0.99 and *P*-value < 0.01. Red and blue lines indicate inverse and positive correlations, respectively.

Since RNA transcription/metabolism and DNA replication/cell cycle were the two thematic biological processes that were markedly modulated in active and latent HIV-1 infections, we sought to identify lncRNAs that were potentially associated with these pathways. For that, we identified the lncRNAs that showed the highest correlations (Pearson correlation coefficient > 0.99 and *P*-value < 0.01) to the transcription and cell cycle-associated mRNAs. This identified 61 lncRNAs with the highest correlations to mRNAs involved in gene transcription (Fig. 6A and C). Similarly, 40 lncRNAs were found to be associated with mRNAs annotated to cell cycle pathways (Fig. 6B and C), indicating the potential roles of these lncRNAs in those cellular pathways. In fact, the lncRNA NRAV has been reported to play roles in the transcription regulation of interferon-stimulated genes, while DLEU2 binds to hepatitis B virus HBx protein to modulate gene transcription (35, 36). Similarly, linc00665 is known to regulate the cell cycle through inhibition of CDKN1C expression, while lncRNA SNHG3 regulates cancer cell proliferation through modulation of CyclinD1 and CDK1 (37, 38). These published reports corroborate the correlative associations of the lncRNAs identified in this study.

### Reversal in expression of latency-associated lncRNAs following reactivation of latently infected cells

**Fig 7:**
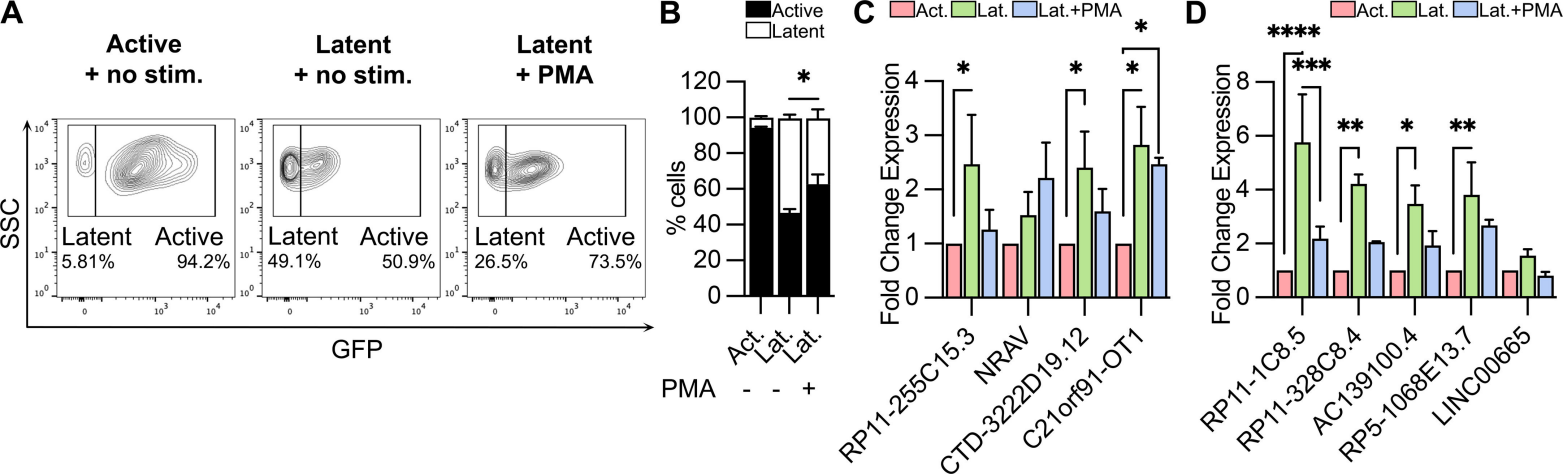
Altered expression of latency-associated lncRNAs following reactivation of latently infected SupT1 cells. (**A**) Representative flow cytometry plots showing proportions of active- (GFP^+^) and latent- (GFP^-^) infected SupT1 cells (gated on mKO2^+^ total infected cells) in flow-sorted active-infected cells without stimulation (left panel), latent-infected cells without stimulation (middle panel), and latent-infected cells with PMA (25 ng/mL) stimulation for 24 h (right panel). (**B**) Proportions of active- (black) and latent- (white) infected SupT1 cells from three independent experiments are presented as mean ± SEM and analyzed by two-way ANOVA test (*P < 0.03; **P < 0.002; ***P < 0.0002; and ****P < 0.0001). (**C and D**) Relative fold changes in the expression of lncRNAs associated to transcription pathway (**C**) and cell cycle pathway (**D**) in latent, unstimulated (green) and latent, PMA-stimulated (blue) cells compared to actively infected, unstimulated (red) SupT1 cells. Expression of lncRNAs from three independent experiments are presented as mean ± SEM, and two-way ANOVA Fisher’s LSD test was used to compare lncRNA expressions from three independent experiments (*P < 0.03; **P < 0.002; ***P < 0.0002; and ****P < 0.0001).

We further investigated how latency-reversing agent (LRA)-mediated stimulation of latently infected SupT1 cells would affect the expression of lncRNAs that were identified as latency-associated and showed correlation to either gene transcription or cell cycle pathways (Fig. 6A through C). Flow-sorted, latently infected SupT1 cells were either treated with a PKC agonist LRA Phorbol myristate acetate (PMA, 25 ng/mL) or left untreated. After 24 h, we observed spontaneous reactivation of viral transcription (GFP expression) in a considerable fraction (mean, 46%) of untreated latently infected SupT1 cells. PMA stimulation led to a further and significant increase in the number of reactivated (mean, 63%) cells (Fig. 7A and B). At 24 h post-stimulation, we harvested the PMA-treated or untreated cells, extracted total cellular RNA, and analyzed the expressions of four transcription-associated lncRNAs and five cell cycle-associated lncRNAs that were significantly over-expressed in latent HIV-1 infection (Fig. 6A through C). While the expression of the transcription-associated lncRNAs, RP11-255C15.3 and CTD-3222D19.12, showed a downregulation after PMA stimulation, the expression of C21orf91-OT1 and NRAV were either unchanged or increased, respectively, compared to latent infection (Fig. 7C). This contrasts with cell cycle-related lncRNAs, which were significantly upregulated in latent infection, showed considerable reductions in their expression following PMA stimulation, including a significant decrease in the expression of the lncRNA RP11-1C8.5 (Fig. 7D). These data show a pattern in lncRNA expression that is specific to HIV-1 infection state and are readily modulated upon changes in the state of infection.

## DISCUSSION

HIV-1 infection is associated with a multitude of changes in cellular functions that contribute to the establishment of either productive or latent infection. An in-depth understanding of such alterations is instrumental for crafting effective therapeutics against HIV-1. The overall aim of this study was an integrative and comparative analysis of the epigenomic and transcriptomic changes that occur in active and latently HIV-1-infected T cells. Several studies have previously investigated alterations in genomic, transcriptomic, or proteomic profiles of HIV-1-infected T cells, often focused on either active- or latently infected cells (16, 17, 39). However, in many instances, the analysis was confounded by the use of a heterogeneous mix of infected cells as the source material, without purification of active- and latently infected cells. We used a well-established HIV-1 latency model of infection of SupT1 cells with a dual reporter virus, HIV_GKO_, that allows specific isolation and analysis of HIV-1 active- or latently infected cells in utmost purity (30). Although we used the SupT1 cell line, it provides an advantage of cellular homogeneity to identify active and latent viral infection-induced changes through bulk transcriptomic and epigenomic analysis. It also facilitates the investigation of cellular reprogramming at the early stages of active and latent infections without any extrinsic stimulations. We employed a multi-omics approach encompassing both ATAC-seq and RNA-seq and directly compared flow-sorted active- or latently infected SupT1 cells to uninfected ones for a more comprehensive insight. In congruence with previous findings, our results show that both epigenetic and transcriptomic profiles of HIV-1-infected T cells are quite distinctive to the state of viral infection and further expand the findings to demonstrate that the magnitude of changes is significantly greater in latent than active infection. We also demonstrated an opposing relationship between chromatin reorganization and gene expression in the two states of HIV-1 infection.

The two most impacted cellular pathways that distinguished active and latent HIV-1 infections of SupT1 cells were gene transcription and cell cycle. HIV-1 infection, specifically the accessory protein, Vpr, is known to modulate the cell cycle (33, 34). Here, we also observed a downregulation of genes enriched in the cell cycle pathway in actively infected cells. However, the inhibition of gene expression and blockade in the cell cycle pathway was significantly higher in latent infection (Fig. 4I). This suggests a possible Vpr-independent mechanism of the cell cycle arrest in HIV-1 latent infection of T cells. The inhibition of cell cycle observed in latently infected cells is consistent with the fact that HIV-1 transcriptional silencing and latency in T lymphocytes are facilitated by the transition of virus-infected, activated CD4^+^ T cells into resting, memory T cells (40). Furthermore, experimental induction of cellular quiescence and restriction of the cell cycle are also known to promote HIV-1 latency in T cells (41). However, the blockade in the cell cycle pathway in latently infected SupT1 cells observed in our study occurred in the absence of immunological memory development or any external coercing factors, indicating an alternate regulatory pathway. Delineation of the exact mechanism of this restriction needs further investigation.

In this study, we defined the expressed lncRNA repertoire in SupT1 cells. Almost 16% of all expressed RNA transcripts in SupT1 cells were lncRNAs (Fig. 2A). We also investigated the effects of HIV-1 infection on lncRNA expression and identified putative functions of the differentially expressed lncRNAs in HIV-1 replication and latency. Indeed, integrated analysis of the epigenetic and transcriptomic profiles of lncRNAs in active- and latently HIV-1-infected T cells had so far remained unexplored. Our study revealed a dramatic impact of HIV-1 infection on both the chromatin accessibility and expression of lncRNAs, which was more pronounced than mRNAs. The distinct expression patterns of lncRNAs in active and latent infections provided evidence that, similar to mRNAs, lncRNAs can also accurately reflect the state of HIV-1 infection in T cells. Furthermore, we showed that LRA, i.e., PMA-mediated reactivation, affects the expression of latency-associated lncRNAs. We observed spontaneous reactivation in a considerable fraction of flow-sorted, latently infected SupT1 cells. It is known that HIV-1 latently infected cells remain in a pseudo-steady state, oscillating between viral transcriptional silence and low level of viral reactivation. The higher level of spontaneous reactivation in SupT1 cells observed in our study could be due to extraneous activation during the long flow-sorting period, although stimulation with PMA further increased the proportion of reactivated cells. Despite the spontaneous reactivation in untreated cells, expression of the lncRNAs examined showed a trend reversal in the PMA-stimulated cells. Significant downregulation in the expression of the lncRNAs in PMA-reactivated cells further suggests a potential role of the lncRNAs in the regulation of HIV-1 replication and/or latency. However, it remains undetermined how other classes of LRAs such as HDAC inhibitors may modulate the expression of the lncRNAs. Future studies on the mechanism of functions of the lncRNAs identified here could identify additional routes for manipulating HIV-1 infection and latency.

In summary, the multi-omics analysis in this study provides new insights into the distinct epigenomic and transcriptional states of active and latent HIV-1-infected T cells, wherein HIV-1 latency has a greater impact than active infection. Knowledge of the discrete cellular states that are conducive to active and latent infections may support the development of novel therapeutic interventions for HIV-1 infection.

## MATERIALS AND METHODS

### Cell lines and culture

HEK-293T and SupT1 cells were obtained from ATCC and TZM-bl cells from the NIH HIV Reagent Program. HEK-293T/TZM-bl and SupT1 cells were cultured in Dulbecco's Modified Eagle Medium (DMEM) or Roswell Park Memorial Institute (RPMI) media, respectively, supplemented with 10% Fetal bovine serum (FBS) and 1% penicillin-streptomycin-glutamine.

### Virus production

HIV_GKO_ plasmid was obtained from Addgene (Catalog#112234). The viral stock was generated by co-transfecting HEK-293T cells with HIV_GKO_ and VSV-G envelope plasmids using Lipofectamine 3000 reagent (Invitrogen). Culture supernatant was harvested 48 h post-transfection, centrifuged, and filtered (0.45 µm) before storage at −80°C in aliquots. The viral stock was titered by infection and X-Gal staining TZM-bl cells.

### SupT1 infection and sorting

SupT1 cells were infected with HIV_GKO_ virus (MOI = 0.1) by spinoculation (3,000 rpm, 30 min, 30°C). Virus-infected or uninfected cells were cultured for 4 days at 37°C with 6% CO2. Uninfected or HIV-1 active-infected (mKO2^+^GFP^+^) and latent-infected (mKO2^+^GFP^-^) cells were flow-sorted using a Biorad S3 cell sorter. Total RNA was extracted from flow-sorted cells using TRIzol reagent (Invitrogen) according to the manufacturer’s protocol, followed by treatment with DNase (Turbo-DNAse-free kit, Invitrogen).

### RNA-seq library preparation and sequencing

Ribosomal RNA was removed from the total RNA (50 ng) using the NEBNext rRNA Depletion kit v2 (Human/Mouse/Rat, NEB), according to the manufacturer’s instructions. The rRNA-depleted samples were used for stranded library preparation using NEBNext UltraII Directional RNA Library Prep Kit for Illumina. The libraries were sequenced on a HiSeq2500 (Illumina) instrument with a targeted average output of 40 million paired-end reads (150 bp) per sample.

### RNA-seq data analysis

Raw RNA-seq reads were first analyzed with FastQC for quality control and then processed through Trim Galore for the removal of low-quality reads and the Illumina sequencing adapters (42). The trimmed, high-quality reads were aligned to the Ensembl human reference genome (GRCh38 v86) using the STAR aligner software (43). Raw read counts for each gene were obtained using -quantmode of GeneCounts. Aligned reads were annotated in accordance with the ENSEMBL database, and the categorization of an RNA transcript as lncRNA was based on the ENSEMBL/GENCODE biotype classifications. RNA transcripts with counts per million mapped reads (cpm) greater than 1 in at least 50% of the samples were considered detectable. Differential expression analysis was carried out using the standard EdgeR pipeline in R (44). For pathways’ analysis, gene lists were pre-ranked based on their fold changes and *P*-values [sign(log2FC) × -log10(*P*-value)] prior to analysis by GSEA using the Reactome database of canonical pathways. WGCNA was performed using the “unsigned” mode following the previously published method (45).

### ATAC-seq library preparation and sequencing

ATAC-seq libraries were prepared from the flow-sorted cells following published protocols (46–48). Briefly, 100,000 cells were used for transposition reaction (Illumina Tagment DNA TDE1 Enzyme and Buffer Kit). Tagmented DNA was purified with MinElute purification kit (Qiagen) and then used for subsequent library preparation by PCR amplification using unique dual-indexing (Illumina Nextera i5 common adapter and i7 index adapter) primers. ATAC-seq libraries were purified using AMPure SPRI beads (Beckman Coulter) and then sequenced on a NextSeq2000 (Illumina) to a targeted, average depth of 50 million, 100 bp paired-end reads per sample.

### ATAC-seq data analysis

Raw ATAC-seq reads were first processed by FastQC and Cutadapt for quality control and adapter trimming (42, 49). After aligning the reads to human reference genome GRCh38 using BWA-MEM, the output data were analyzed by SAMStat for quality control and sorted by SAMtools (50–52). Post-alignment filtering was performed by SAMtools, “MarkDuplicates” program of Picard tools and ENCODE ATAC-seq script assign_multimappers.py to remove PCR duplicates, secondary alignments, and unmapped reads. Quality control of fragment size distribution was carried out by ATACseqQC (53). To correct for the insertion of two adapters, reads aligned to the positive strand were shifted 4 bp downstream and those aligned to the negative strand were shifted 5 bp upstream. The processed reads were used for all subsequent analyses. Chromatin-accessible peaks were identified by MACS2 by executing ENCODE script encode_macs2_atac.py (smooth_win = 150, pval_thresh = 0.01, gensz = 3.21e^9^) (54). Irreproducible discovery rate (IDR) filtering of pooled FASTQ files for each group was determined through the same pipeline described above. For each group, the IDR framework was applied to select highly reproducible peaks (IDR < 0.05) between individual replicates and the pooled data of the respective group. The selected peaks from all replicates of a group were then merged by BEDTools as the final set of IDR peaks. PCA was performed on peaks with variance among samples (IQR filtering was applied) by using FactoMineR. All the peaks were annotated to the nearest genes based on ENSEMBL annotation package “EnsDb.Hsapiens.v86” by ChIPseeker (55). To perform differential peak analysis, we merged IDR peaks from all the groups and counted the reads that overlap merged peaks for each sample. Differentially accessible regions between groups were then determined using EdgeR pipeline in R (44).

### Quantitative RT-PCR

For relative quantification, about 25 ng of DNA-free total RNA for each lncRNA was reverse-transcribed using iTaq cDNA Synthesis Kit (Bio-Rad) and then analyzed with ABI 7500 Fast Real-time PCR System (Applied Biosystem) using iTaq Universal SYBR Green Supermix (Bio-Rad). The expression of lncRNAs was normalized based on three house-keeping genes: GAPDH, 18S rRNA, and U6. Primer sequences are available in Table S1.

### Statistical analysis

Gene expression between groups (normalized log counts from RNA-seq or fold change from qRT-PCR) was compared with a two-way ANOVA Fisher’s LSD test. A *P*-value < 0.05 was considered significant. The distribution of frequencies of lncRNA subclasses between groups was compared with Chi-square test. GraphPad Prism software was used for statistical analysis.

## Data Availability

Data accessible from GEO: GSE212462 (ATAC-seq) and GSE212463 (RNA-seq).

## SUPPLEMENTAL MATERIAL

Supplemental Material mbio.02619-23-s0001.pdf Primer sequences.

ASM does not own the copyrights to Supplemental Material that may be linked to, or accessed through, an article. The authors have granted ASM a non-exclusive, world-wide license to publish the Supplemental Material files. Please contact the corresponding author directly for reuse.
