# Supplementary material for: HIV-1 active and latent infections induce disparate chromatin reorganization and transcriptional regulation of mRNAs and lncRNAs in SupT1 cells
Source: mBio. 2023 Dec 1;14(6):e02619-23. doi: 10.1128/mbio.02619-23 (PMC10746154; doi:10.1128/mbio.02619-23)
Supplement: Supplemental Material — Primer sequences. [file mbio.02619-23-s0001.pdf]

Table S1: Primers sequences used for quantitative RT-PCR.

Gene name	qRT-PCT Primer sequence
RP11-1C8.5-QF<br>RP11-1C8.5-QR	CAGCCTTATTCTGAGGACCATAC<br>GCCAAATTGCCTAACCTCTTTC
RP11-328C8.4-QF<br>RP11-328C8.4-QR	CCACTGGCTGTGAGTTCAATA<br>GCAGTTTCATCAACCCATCAATAC
AC139100.4-QF<br>AC139100.4-QR	CTGGAAAGGACTCGAAGACAAA<br>GCTGGGTTGAGAGATGTGATG
RP5-1068E13.7-QF<br>RP5-1068E13.7-QR	TTATGCTAGGTGGAGAGGTAGG<br>TGCGTGTCTCTAACGAGTTTC
LINC00665-QF<br>LINC00665-QR	CACAGCAAGCCCCTGGAT<br>CAGATACTCAAGATGGGTGGTG
RP11-255C15.3-QF<br>RP11-255C15.3-QR	GAGCCCTCATGAATGGGATTAG<br>TTGCAGACTGCTGACTTCTC
NRAV-QF<br>NRAV-QR	GCTGTCTGGAGAGATGAAGAAA<br>CATCCCAGCTCTGTCACTTT
CTD-3222D19.12-QF<br>CTD-3222D19.12-QR	GATGCCTGTAATCCCATCTACTT<br>CACGACTTTGGCTCACTGTA
C21orf91-OT1-QF<br>C21orf91-OT1-QR	AATGTTTGGATGGCACAAGTC<br>CTCCTATCCAGTTTCTTTGCTTTAC
NEAT1-QF<br>NEAT1-QR	CCAGTTTTCCGAGAACCAAA<br>ATGCTGATCTGCTGCGTATG
GAPDH-QF<br>GAPDH-QF	GACAAGCTTCCCGTTCTCAG<br>GAGTCAACGGATTTGGTCGT
U6-QF<br>U6-QR	CTCGCTTTGGCAGCACA<br>AACGCTTCACGAATTTGCGT
18SrRNA-QF<br>18SrRNA-QR	GGCCCTGTAATTGGAATGAGTC<br>CCAAGATCCAACTACGAGCTT
